# Inferring cancer disease response from radiology reports using large language models with data augmentation and prompting

**DOI:** 10.1093/jamia/ocad133

**Published:** 2023-07-14

**Authors:** Ryan Shea Ying Cong Tan, Qian Lin, Guat Hwa Low, Ruixi Lin, Tzer Chew Goh, Christopher Chu En Chang, Fung Fung Lee, Wei Yin Chan, Wei Chong Tan, Han Jieh Tey, Fun Loon Leong, Hong Qi Tan, Wen Long Nei, Wen Yee Chay, David Wai Meng Tai, Gillianne Geet Yi Lai, Lionel Tim-Ee Cheng, Fuh Yong Wong, Matthew Chin Heng Chua, Melvin Lee Kiang Chua, Daniel Shao Weng Tan, Choon Hua Thng, Iain Bee Huat Tan, Hwee Tou Ng

**Affiliations:** Division of Medical Oncology, National Cancer Centre Singapore, Singapore; Duke-NUS Medical School, Singapore; Department of Computer Science, National University of Singapore, Singapore; Division of Medical Oncology, National Cancer Centre Singapore, Singapore; Department of Computer Science, National University of Singapore, Singapore; Institute of Systems Science, National University of Singapore, Singapore; Institute of Systems Science, National University of Singapore, Singapore; Institute of Systems Science, National University of Singapore, Singapore; Institute of Systems Science, National University of Singapore, Singapore; Division of Medical Oncology, National Cancer Centre Singapore, Singapore; Duke-NUS Medical School, Singapore; Division of Medical Oncology, National Cancer Centre Singapore, Singapore; Division of Medical Oncology, National Cancer Centre Singapore, Singapore; Division of Radiation Oncology, National Cancer Centre Singapore, Singapore; Division of Radiation Oncology, National Cancer Centre Singapore, Singapore; Division of Medical Oncology, National Cancer Centre Singapore, Singapore; Duke-NUS Medical School, Singapore; Division of Medical Oncology, National Cancer Centre Singapore, Singapore; Duke-NUS Medical School, Singapore; Division of Medical Oncology, National Cancer Centre Singapore, Singapore; Duke-NUS Medical School, Singapore; Duke-NUS Medical School, Singapore; Department of Diagnostic Radiology, Singapore General Hospital, Singapore; Division of Radiation Oncology, National Cancer Centre Singapore, Singapore; Yong Loo Lin School of Medicine, National University of Singapore, Singapore; Duke-NUS Medical School, Singapore; Division of Radiation Oncology, National Cancer Centre Singapore, Singapore; Data and Computational Science Core, National Cancer Centre Singapore, Singapore; Division of Medical Oncology, National Cancer Centre Singapore, Singapore; Division of Clinical Trials and Epidemiological Sciences, National Cancer Centre Singapore, Singapore; Duke-NUS Medical School, Singapore; Division of Oncologic Imaging, National Cancer Centre Singapore, Singapore; Division of Medical Oncology, National Cancer Centre Singapore, Singapore; Duke-NUS Medical School, Singapore; Data and Computational Science Core, National Cancer Centre Singapore, Singapore; Department of Computer Science, National University of Singapore, Singapore

**Keywords:** large language models, natural language processing, cancer, radiology, healthcare

## Abstract

**Objective:**

To assess large language models on their ability to accurately infer cancer disease response from free-text radiology reports.

**Materials and Methods:**

We assembled 10 602 computed tomography reports from cancer patients seen at a single institution. All reports were classified into: no evidence of disease, partial response, stable disease, or progressive disease. We applied transformer models, a bidirectional long short-term memory model, a convolutional neural network model, and conventional machine learning methods to this task. Data augmentation using sentence permutation with consistency loss as well as prompt-based fine-tuning were used on the best-performing models. Models were validated on a hold-out test set and an external validation set based on Response Evaluation Criteria in Solid Tumors (RECIST) classifications.

**Results:**

The best-performing model was the GatorTron transformer which achieved an accuracy of 0.8916 on the test set and 0.8919 on the RECIST validation set. Data augmentation further improved the accuracy to 0.8976. Prompt-based fine-tuning did not further improve accuracy but was able to reduce the number of training reports to 500 while still achieving good performance.

**Discussion:**

These models could be used by researchers to derive progression-free survival in large datasets. It may also serve as a decision support tool by providing clinicians an automated second opinion of disease response.

**Conclusions:**

Large clinical language models demonstrate potential to infer cancer disease response from radiology reports at scale. Data augmentation techniques are useful to further improve performance. Prompt-based fine-tuning can significantly reduce the size of the training dataset.

## BACKGROUND AND SIGNIFICANCE

The volume of healthcare data produced in oncology has increased tremendously.^1^^,^^2^ However, much of this clinical data still remains in free text, which has limited the speed at which real-world insights can be discovered to improve and advance patient care and research.^3^

Natural language processing (NLP), defined as the automated processing of natural language texts, has the potential to provide a solution for large-scale information extraction and inference of key variables within clinical texts.^4^ Deep learning-based models have achieved state-of-the-art performance on various NLP tasks,^5^ and have many uses in oncology such as case identification and outcome determination.^6^^,^^7^

Progression-free survival is a key clinically meaningful endpoint that has supported the regulatory approval of many anticancer therapies.^8^^,^^9^ Real-world evidence (RWE), often consisting of endpoints gathered in electronic health records (EHRs), is playing an increasingly important role in healthcare decisions.^10^ Prior work has explored multiple methods for curating real-world progression-free survival from EHRs and has shown this to reliably correlate with overall survival as well as Response Evaluation Criteria in Solid Tumors (RECIST).^11^^,^^12^ In these methods, radiology reports were manually reviewed by human annotators for disease response, which was then used to derive progression-free survival.^13^ We illustrate how this can be done in Supplementary Figure S1.

Prior work had used a convolutional neural network (CNN) to ascertain disease response from radiology reports as a basis for progression-free survival.^14–16^ In the years since, a succession of transformer models have achieved state-of-the-art performance on many NLP benchmarks.^17–25^ Applying them to this task of inferring cancer disease response can be helpful to researchers in the analysis of large cancer datasets. Such models can also serve as a clinical decision support tool by providing an automated second opinion of disease response for radiology reports.

## OBJECTIVE

We aim to: (1) evaluate the relative performance of newer large language models against previous NLP methods for cancer disease response classification; (2) explore the effects of data augmentation techniques and prompt-based fine-tuning on model performance; and (3) test the best-performing model on a set of RECIST-based classifications to explore its potential utility in clinical trials. We believe that these models will be useful to clinicians and researchers for clinical research and clinical decision support.

## MATERIALS AND METHODS

### Data source and study participants

Deidentified radiology reports were retrieved for cancer patients of all stages seen at the National Cancer Centre Singapore (NCCS) who were also recruited into prospectively maintained research databases on colorectal, lung, breast, and gynaecological cancer. Radiology reports retrieved were all in English. They were reviewed to include only computed tomography (CT) scans which had complete reports, had prior comparison CT scans, reported cancer response assessments which were not indeterminate and spanned multiple anatomical regions. For this study, we define CT scans involving multiple anatomical regions as the following scans: chest-abdomen, chest-abdomen-pelvis, neck-chest-abdomen, neck-chest-abdomen-pelvis, brain-neck-chest, brain-neck-chest-abdomen, brain-neck-chest-abdomen-pelvis, brain-paranasal space-neck-chest, brain-paranasal space-neck-chest-abdomen, and brain-paranasal space-neck-chest-abdomen-pelvis. These were selected as they reflect our institutional practice of using scans involving multiple anatomical regions for restaging of cancer patients. We collected disease characteristics primary site and stage as well as demographic information including age, gender, and ethnicity. However, we did not collect socioeconomic status of patients for this study. Further details on the inclusion criteria can be found in Supplementary Material.

We obtained permission from the principal investigator and NCCS division of clinical trials and epidemiological sciences to retrospectively retrieve deidentified radiology reports from an investigator-initiated clinical trial in ovarian cancer (NCT02736305) together with their accompanying RECIST responses. These were used as an external validation cohort for our best-performing model.

### Report curation and outcome definitions

All retrieved radiology reports were screened by a research data management team (HJT, FLL) under the supervision of a medical oncologist (RSYCT) and included the date when the radiology study was performed, study modality and region, as well as the full radiology report. Each full radiology report included 2 sections: “Report” which was the detailed radiology description and “Conclusion” which summarized the main findings. The selected reports were reviewed and annotated for disease response by a team of medical research staff (MIBI, WWL) and separately by a medical oncologist (RSYCT). These annotations were done independently and blindly according to consensus classification guidelines (Supplementary Table S1) developed by 2 medical oncologists (RSYCT and WCT) in consultation with a senior radiologist (CHT). These guidelines were adapted from a previous study.^26^ Reports with indeterminate findings or where a disease response could not be assigned with certainty by human annotators were excluded. Where there was disagreement between the first 2 sets of annotations (MIBI/WWL and RSYCT), a curator medical oncologist (WCT) would check and make the final decision on the assigned class. This final decision would be used for the gold standard set to train and test the NLP models, while the 2 prior sets of annotations were used to measure the inter-annotator agreement (IAA). IAA was evaluated using Cohen’s kappa statistic *κ* (Supplementary Figure S2).

### Model training and evaluation

We randomly divided the annotated radiology reports at the patient level into training (80%), development (10%), and test sets (10%) while preserving representation of all 4 tumor types. We chose patient-level randomization as models are expected to be tested on new patients without any prior reports when deployed in clinical workflows. The conclusion section of a radiology report was used as the input, while disease response was used as the target output for this classification task in our experiments.

For the conventional machine learning models, we used a linear support vector machine classifier (linearSVC) as well as an ensemble voting model built with a logistic regression (LR) model, an extreme gradient boosting (XGBoost) model, and a linearSVC model. These models process the raw text using term frequency-inverse document frequency (TF-IDF). The models were implemented in Python using scikit-learn.^27^

For the bidirectional long short-term memory (Bi-LSTM) model, the training data were first tokenized using the Keras tokenizer and padded with zeros to the maximum sequence length. The model was developed using Keras.^28^ The first layer was an embedding layer with input dimension equal to the vocabulary size and an output size of 64. This was followed by the bi-directional LSTM layers and 2 fully connected dense layers containing 64 nodes and 4 nodes, respectively. Hyperparameter tuning was performed on the number of epochs, learning rate, and batch size.

The CNN model was developed using Tensorflow and Keras.^28^ We treated each radiology report as a sequence of tokens embedded into a vector space and then fed it into a 1-dimensional convolutional layer before reducing the dimension using max-pooling. The pooling layer output was then passed to fully connected layers to model global interactions among the input words.

Transformer models were implemented using the Hugging Face Transformers library and PyTorch framework.^29^^,^^30^ Our selected list of transformer models was: BERT (Bidirectional Encoder Representations from Transformers), BioBERT (BERT for Biomedical Text Mining), BioClinicalBERT (Clinical BioBERT), BioMegatron, DeBERTa (Decoding-enhanced BERT with disentangled attention), GatorTron, PubMedGPT (PubMed Generative Pre-trained Transformer), RadBERT (BERT-based language model adapted for radiology), RoBERTa (Robustly optimized BERT approach) and XLNet. For each model, we experimented with various model hyperparameters including the number of epochs, learning rate, training batch size, evaluation batch size, and maximum sequence length.

Model accuracy was the chosen performance metric. As this was a multiclass classification task, accuracy is equivalent to micro-F1, microprecision, and microrecall score.^31^

### Data augmentation

We observed that sentences in the conclusion section of a radiology report are relatively independent. As such, changing the order of these sentences does not significantly affect the coherence of the conclusion text. Hence, we used sentence permutation to generate new synthetic radiology reports to augment our training data and increase the number of training samples. For each conclusion text in the training set, we produce *P* distinct randomly permuted conclusion texts as augmented data. The label (ie, disease response) of a conclusion text stays unchanged after sentence permutation. *P* was set to 10 in our experiments.

During training, we obtained the output label distribution ${\hat{y}}_{P}$ of a permuted sample. We compared the output label distribution $\hat{y}$ of the original sample and ${\hat{y}}_{P}$ by mean-squared error (MSE) to produce a consistency loss: $L_{\mathrm{MSE}}=\frac{1}{C}{\left(\hat{y}-{\hat{y}}_{P}\right)}^{2}$, where C=4 (the number of disease responses). The overall loss function L is the weighted sum of cross entropy loss $L_{\mathrm{CE}}$ and consistency loss $L_{\mathrm{MSE}}$: $L={\alpha L}_{\mathrm{CE}}+\beta L_{\mathrm{MSE}}$. α and β were set to 1 and 10, respectively. Our data augmentation approach is similar to the unsupervised data augmentation method proposed by Xie et al,^32^ but we used sentence permutation instead of back translation to generate our augmented data.

### Prompt-based fine-tuning

The prompt-based fine-tuning method we used was ADAPET,^33^ which uses a pre-trained language model (PLM), such as the GatorTron model in our work. The input to ADAPET was a cloze-style prompt with a [MASK] token (or tokens) to the PLM, and the output was a probability distribution produced by the PLM over tokens to be predicted for [MASK], which then corresponded to class labels via a verbalizer. For training, ADAPET fine tunes the PLM by minimizing the sum of a decoupled label loss and a label-conditioned masked language model loss.

In our ADAPET experiments, the prompt was “*[INPUT_TEXT] [SEP] In summary, this is a [MASK]*,” where [INPUT_TEXT] was the conclusion text from a radiology report and [SEP] was a special separator token. The verbalizer is a function that maps each actual class label to the tokens to be predicted for [MASK]. The verbalizer we used was {“0”: “no evidence of disease”, “1”: “partial response”, “2”: “stable disease”, “3”: “progressive disease”}. Hyperparameters used during training followed the default values of ADAPET, except that num_batches was 2000, lr was 1*e*−5, eval_every was 100, and max_num_lbl_tok was 2.

## RESULTS

### Cohort description

**Figure 1.**
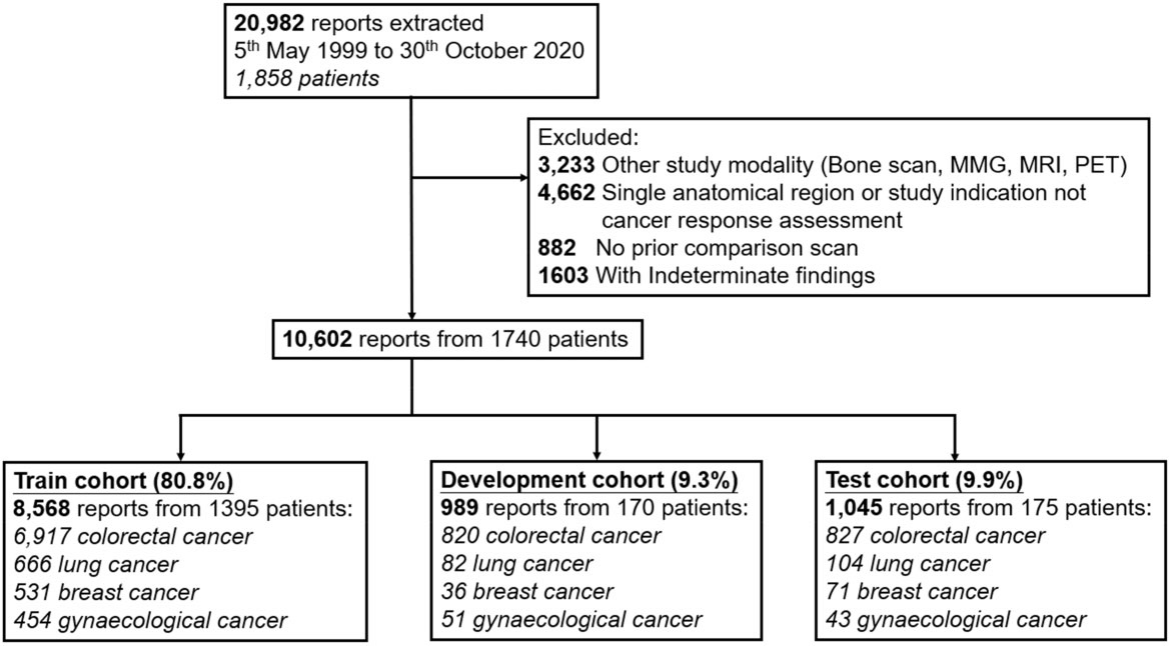
Participant flow diagram. MMG: mammography; MRI: magnetic resonance imaging; PET: positron emission tomography.

The participant flow diagram is presented in Figure 1. The average number of radiology reports curated per patient was 6. Dataset characteristics are shown in Table 1. The IAA between annotators, measured by Cohen’s kappa statistic, was high (*κ* = 0.83). The distribution of human-annotated cancer disease responses for the training, development, and test sets is given in Table 2.

**Table 1.** Dataset characteristics

	Training set (%)	Development set (%)	Test set (%)	Total (%)
Number of patients	1395 (100.0)	170 (100.0)	175 (100.0)	1740 (100.0)
Primary site of cancer of patient				
Colorectal	1120 (80.3)	139 (81.8)	137 (78.3)	1396 (80.2)
Breast	61 (4.4)	4 (2.4)	11 (6.3)	76 (4.4)
Gynaecological	105 (7.5)	14 (8.2)	10 (5.7)	129 (7.4)
Lung	109 (7.8)	13 (7.6)	17 (9.7)	139 (8.0)
Stage at diagnosis				
Early (I–III)	982 (70.4)	44 (25.9)	50 (28.6)	1076 (61.8)
Metastatic (IV)	413 (29.6)	126 (74.1)	125 (71.4)	664 (38.2)
Sex				
Male	733 (52.5)	92 (54.1)	96 (54.9)	921 (53.0)
Female	662 (47.5)	78 (45.9)	79 (45.1)	819 (47.0)
Age				
<50	210 (15.0)	29 (17.0)	27 (15.4)	266 (15.3)
50–59	410 (29.4)	47 (27.6)	46 (26.3)	503 (28.9)
60–69	508 (36.4)	55 (32.4)	63 (36.0)	626 (36.0)
70–79	234 (16.8)	32 (18.9)	32 (18.3)	298 (17.1)
>80	33 (2.4)	7 (4.1)	7 (4.0)	47 (2.7)
Race and ethnicity				
Chinese	1172 (84.0)	153 (90.0)	154 (88.0)	1479 (85.0)
Malay	113 (8.1)	7 (4.1)	8 (4.6)	128 (7.4)
Indian	44 (3.2)	1 (0.6)	8 (4.6)	53 (3.0)
Other^a^	66 (4.7)	9 (5.3)	5 (2.8)	80 (4.6)

a Other races and ethnicities in our dataset include: Bangladeshi, Burmese, Eurasian, Filipino, Indonesian, Japanese, Korean, Nepalese, Pakistani, Sikh, Sri Lankan, Vietnamese, and White.

**Table 2.** Human-annotated cancer disease response by report

Response	Training set (%)	Development set (%)	Test set (%)	Total (%)
Total	8568 (100.0)	989 (100.0)	1045 (100.0)	10 602 (100.0)
NED	3342 (39.0)	380 (38.4)	411 (39.3)	4133 (39.0)
PR	994 (11.6)	103 (10.4)	107 (10.2)	1204 (11.4)
SD	1230 (14.4)	134 (13.5)	167 (16.0)	1531 (14.4)
PD	3002 (35.0)	372 (37.6)	360 (34.4)	3734 (35.2)

*Abbreviation*: NED: no evidence of disease; PR: partial response; SD: stable disease; PD: progressive disease.

### Model performance

The performance of the different classification models is summarized in Table 3. All accuracies (for both “Base Model” and “With sentence permutation and consistency loss”) reported in Table 3 for the GatorTron, BioMegatron, BioBERT, RoBERTa, RadBERT, XLNet, PubMedGPT, DeBERTa, BioClinicalBERT, BERT, CNN, and BiLSTM models were based on averaging 3 runs with different random seeds. All base transformer models achieved significantly better performance than other model types, with accuracy ranging from 0.8708 to 0.8916, compared with the best-performing Bi-LSTM model which achieved accuracy of 0.8166. The best-performing model was the GatorTron model which achieved an accuracy of 0.8916. The breakdown of performance of this model by category of disease response is shown in Supplementary Table S2. Our RECIST-based external validation set of 37 reports had a high correlation between RECIST response and curated disease response (Spearman’s *ρ* = 0.85). When validated on the RECIST-based dataset, this model achieved an accuracy of 0.8919.

**Table 3.** Results of various models on the test set as well as after data augmentation (with sentence permutation and consistency loss) for disease response classification

Model	Base model	With sentence permutation and consistency loss
	Accuracy^a^	Accuracy^a^
GatorTron	0.8916	0.8976
BioMegatron	0.8861	0.8935
BioBERT	0.8817	0.8897
RoBERTa	0.8813	0.8868
RadBERT	0.8813	0.8867
XLNet	0.8781	0.8842
PubMedGPT	0.8762	0.8772
DeBERTa	0.8746	0.8766
BioClinicalBERT	0.8746	0.8820
BERT	0.8708	0.8762
CNN	0.8319	0.8360
Ensemble voting classifier^b^	0.8278	NE^c^
BiLSTM	0.8166	0.8297
linearSVC	0.8026	NE

a All accuracies reported for GatorTron, BioMegatron, BioBERT, RoBERTa, RadBERT, XLNet, PubMedGPT, DeBERTa, BioClinicalBERT, BERT, CNN, and BiLSTM models were based on averaging 3 runs with different random seeds.

b Ensemble voting classifier (soft vote) based on logistic regression (LR), XGBoost, and linear support vector machine classifier (linearSVC).

c NE: Not evaluated.

Data augmentation further improved the accuracy of the best-performing model to 0.8976. It was also observed that data augmentation improves the accuracy of all models as shown in Table 3. For the GatorTron, BioMegatron, BioBERT, RoBERTa, RadBERT, BioClinicalBERT, BERT, and BiLSTM models, the difference between the accuracy using sentence permutation and consistency loss and the accuracy of the base model was also statistically significant (*P* < .01), based on the paired *t* test. Since the ensemble voting classifier and linearSVC are not based on neural networks, sentence permutation with consistency loss is not directly applicable to them and so we do not report these scores for them.

As shown in Table 4, prompt-based fine-tuning with GatorTron achieved a relatively good accuracy of 0.8609 on this task after training on only 500 reports, while GatorTron without prompt-based fine-tuning had accuracy of only 0.5464 after training on 500 reports. While training on the full training set of 8568 reports with prompt-based fine-tuning achieved an accuracy of 0.8890 (compared with 0.8609 when training on 500 reports), the moderate reduction in accuracy could be achieved by a reduction in training time of 63%. It was also noted in our experiments that the accuracy of 0.8890 achieved by prompt-based fine-tuning with the full training set was marginally worse than the accuracy of 0.8916 achieved by the base GatorTron model using traditional fine-tuning and without using any prompt.

**Table 4.** GatorTron performance and the number of training reports with and without prompt-based fine-tuning

Number of reports in training set	Without prompt-based fine-tuning	With prompt-based fine-tuning
	Accuracy	Accuracy
8568 (full set)	0.8916	0.8890
5000	0.8654	0.8820
1000	0.7607	0.8654
500	0.5464	0.8609
100	0.5435	0.8102

**Figure 2.**
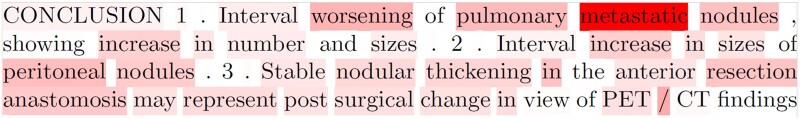
Visualization of attention weights assigned by the GatorTron transformer model to a sample radiology report. A word assigned a higher weight, hence more important, is shaded with a darker red color. This report was accurately classified as progressive disease. PET: positron emission tomography; CT: computed tomography.

**Figure 3.**
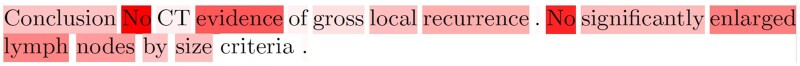
Visualization of attention weights assigned by the GatorTron transformer model to another sample radiology report. A word assigned a higher weight, hence more important, is shaded with a darker red color. This report was accurately classified as no evidence of disease.

Below, we show example visualizations of the weights assigned by the GatorTron transformer model to sample radiology reports in Figures 2 and 3. This provides a level of interpretability for the model’s predictions for human end-users which is important to build confidence in using such models in research or clinical processes.

## DISCUSSION

We developed deep learning-based NLP models that are able to classify cancer disease response in radiology reports with high accuracy. Our best-performing GatorTron model trained on radiology reports achieved high overall performance with an accuracy of 0.8976. This model also performed well on a separate external evaluation set consisting of RECIST-based radiology reports, achieving an accuracy of 0.8919. This NLP tool can be used by researchers to derive progression-free survival in large cancer datasets. This may help to speed up the process of data registry building and uncover new observations such as the effectiveness of cancer drugs in various real-world patient subgroups, providing novel hypotheses for subsequent research (see Supplementary Figure S1). It can also be considered as a clinical decision support tool for clinicians by providing an automated second opinion of disease response for radiology reports. It could also help to highlight reports with disease progression to ordering physicians for earlier attention and review.

Our results demonstrate that transformer models consistently outperformed Bi-LSTM, CNN, and conventional machine learning models by a significant margin. This builds on prior work by Kehl et al,^14^^,^^16^ and establishes large clinical language models as the best-performing NLP method to infer disease progression from radiology reports.

All the pretrained large language models that we used in this work are based on the same architecture—transformer encoder (except PubMedGPT which is based on transformer decoder). They mainly differ in the pretraining objective and pretraining data. For pretraining objective, GatorTron and BioMegatron follow Megatron-LM, while BioBERT, BioClinicalBERT, RoBERTa, and RadBERT follow BERT. GatorTron, BioMegatron, BioBERT, and BioClinicalBERT are pretrained with clinical data; RadBERT is pretrained with radiology reports; and the other models are pretrained with general domain data.

Taking a large language model and further training it (ie, fine-tune) on the full training set of 8568 reports can be done on one Nvidia A100 GPU (40 GB RAM). The training time on the full training set of radiology reports is similar for the different large language models. It takes 20 min for a base model and 60 min with sentence permutation and consistency loss.

GatorTron was our best-performing transformer model. This is consistent with the trend of larger language models achieving better performance.^34^ As a comparison, GatorTron had 345 million parameters compared with BioBERT’s 110 million parameters.^17^^,^^25^ In addition, it was observed that while GatorTron’s incremental gain in accuracy over BioBERT was smaller compared with gains over non-transformer models, model run time for GatorTron was faster than BioBERT (2.5 min vs 11 min) to achieve best results due to the need to run fewer epochs (1 vs 5). This should be considered when building scalable production-ready clinical NLP systems.

Of interest, we also tested the GatorTron model on a RECIST dataset and achieved an accuracy of 0.8919. Although a small dataset, RECIST responses in our dataset did have a high correlation with curated disease response (Spearman’s *ρ* = 0.85). This is in line with Ma et al^12^ (*n* = 1072 from 12 clinical trials) which showed that response assessment based on radiology reports correlates highly with clinical trial RECIST response rates (Spearman’s *ρ* = 0.99). These findings raise the future possibility of using such NLP models in the setting of clinical trials to increase efficacy of trial conduct.

In addition, we demonstrated novel techniques to improve performance of transformer models on our classification task. Firstly, we showed that data augmentation can still help to improve the accuracy of large language models fine-tuned on already sizeable training sets prior to data augmentation. Secondly, we demonstrated that prompt-based fine-tuning was also able to reduce the number of training reports from more than 8000 to 500 and still achieved a relatively good accuracy of 0.8609. Taken together, both techniques can be especially useful in healthcare for NLP tasks where large well-annotated datasets are not common, due to the challenge in assembling large consented cohorts as well as annotating their data.

The advantage of prompt-based fine-tuning is that it is able to achieve a relatively good accuracy with a small number of training examples, because the phrasing of the prompt gives a valuable clue to the model that its task is to predict the cancer disease response class to fill the words at the masked position. Such a clue is particularly useful when there are few training examples for the model to learn the mapping from a radiology report to the cancer disease response class. However, in our experiments, when given the full set of 8568 training examples, prompt-based fine-tuning achieved an accuracy of 0.8890, which was marginally worse than the accuracy of 0.8916 achieved by the base model using traditional fine-tuning and without using any prompt. When many training examples are available, the training examples are sufficient by themselves to allow the model to learn the mapping from a radiology report to the cancer disease response class, and the use of a prompt no longer adds much value. Our finding is also consistent with past research,^35^ which also reported that prompt-based fine-tuning was slightly worse than traditional fine-tuning without any prompt when many training examples were available during training.

Limitations of our study include radiology reports restricted to those involving multiple anatomical regions originating from a single center over a long period, and generalizability of the model to other hospitals remains to be seen. Our RECIST dataset is also small and further studies need to be done to validate our observations. However, the strengths of our study include a high-performance model across 4 cancer types on a large real-world dataset. This shows potential for further multicenter validation studies as well as exploration of utility in clinical trials.

## CONCLUSION

In conclusion, our results demonstrate the feasibility of using deep learning-based NLP models in inferring cancer disease response from radiology reports at scale. Large language models based on transformers consistently outperformed other methods, with GatorTron being the best-performing model for this NLP task. Data augmentation is useful to improve performance while prompt-based fine-tuning can significantly reduce the size of the training dataset.

## Supplementary Material

ocad133_Supplementary_Data

## Data Availability

The data underlying this article will be shared on reasonable requests to the corresponding author. The code for this article is available via GitHub at: https://github.com/nusnlp/cancer-response-inference.
