# Supplementary material for: Inferring cancer disease response from radiology reports using large language models with data augmentation and prompting
Source: J Am Med Inform Assoc. 2023 Jul 14;30(10):1657–64. doi: 10.1093/jamia/ocad133 (PMC10531105; doi:10.1093/jamia/ocad133)

**SUPPLEMENTARY MATERIAL**

**Further Notes on Inclusion Criteria:**

Reports with no prior scans for comparison or if the study indication was not for cancer response assessment were excluded. We also used only the conclusion section of radiology reports for model training and development as we found that training on the full report had lower accuracy, compared to training on only the conclusion sections.

**List of Supplementary Tables:
Table S1.** Overall consensus guidelines on disease response classification

**Table S2.** GatorTron performance (with sentence permutation and consistency loss) by disease response category

**List of Supplementary Figures:
Figure S1.** Using Natural language processing (NLP) to derive progression-free survival

**Figure S2.** Cohen’s kappa statistic as a measure of inter-annotator agreement.

**Table S1.** Overall consensus guidelines on disease response classification

| 1. This indicates the overall cancer disease response compared to prior studies. 2. Tumor size is the primary determinant of status, unless another feature was highlighted as indicating a status change despite stable tumor size. 3. In the absence of specific reference to size, surrogate indicators (e.g., general statements on status, enhancement, and presence of new lesions) were used to determine status. 4. Only changes from the most recent comparison study were considered. 5. Each report must be assigned one of four disease response categories below:  \| **Response** \| **Description** \| \| --- \| --- \| \| No evidence of disease (NED) \| All tumors have resolved when compared to the previous scan. \| \| Partial response (PR) \| All tumors are smaller compared to the previous scan.  Some tumors remain the same size while other tumors are smaller compared to the previous scan.  Even terms such as ‘marginal decrease’ or ‘slight decrease in prominence’ would be considered a decrease in size and thus response. \| \| Stable disease (SD) \| All tumors remain the same size compared to the previous scan. \| \| Progressive disease (PD) \| All tumors are larger compared to the previous scan.  Some tumors remain the same size while other tumors are larger compared to the previous scan.  Some tumors are smaller but other tumors are larger compared to the previous scan.  Even terms such as ‘marginal increase’ or ‘slight increase in prominence’ would be considered an increase in size and thus progression. \| |
| --- | --- | --- | --- | --- | --- | --- | --- | --- | --- | --- |

Note: The above consensus guidelines were adapted from a previous study by Cheng LTE et al *J Digit Imaging*. 2010;23(2):119-132.

**Table S2.** GatorTron performance (with sentence permutation and consistency loss) by disease response category. NED: no evidence of disease; PR: partial response; SD: stable disease; PD: progressive disease.

|  | Precision | Recall | Accuracy | Support |
| --- | --- | --- | --- | --- |
| NED | 0.9095 | 0.9538 | 0.9311 | 411 |
| PR | 0.8000 | 0.8972 | 0.8458 | 107 |
| SD | 0.8613 | 0.7066 | 0.7763 | 167 |
| PD | 0.9300 | 0.9222 | 0.9261 | 360 |

**Figure S1.** Using Natural language processing (NLP) to derive progression-free survival


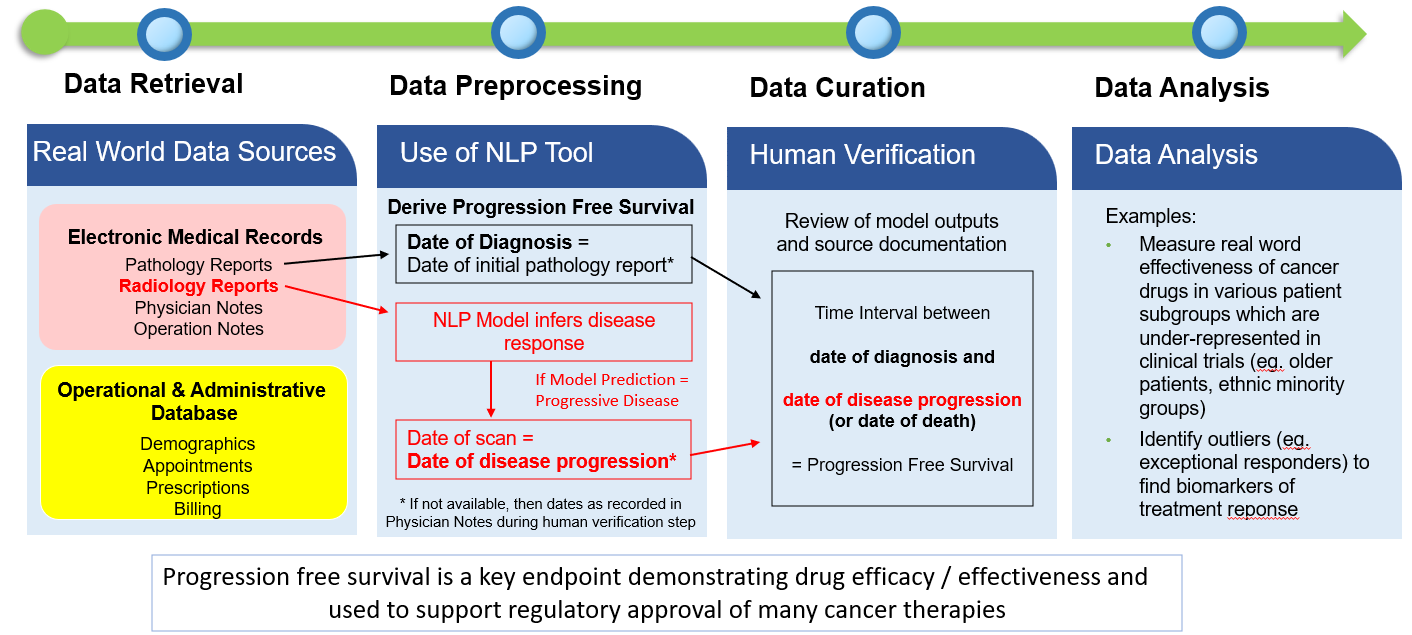


**Figure S2.** Cohen’s kappa statistic as a measure of inter-annotator agreement.


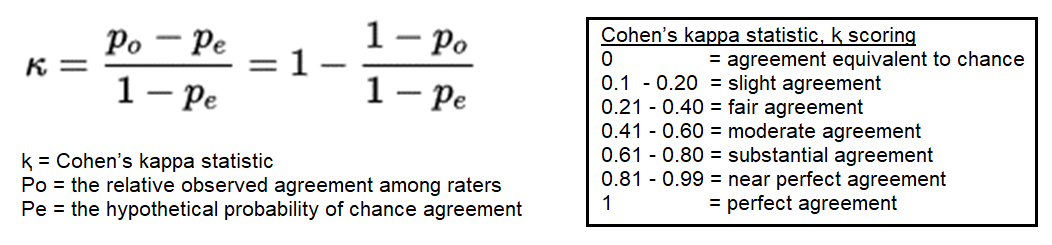

Supplement: ocad133_Supplementary_Data [file ocad133_supplementary_data.docx]
